# Increasing the Radioiodine Dose Does Not Improve Cure Rates in Severe Graves' Hyperthyroidism: A Clinical Trial with Historical Control

**DOI:** 10.1155/2013/958276

**Published:** 2013-07-31

**Authors:** Jose Miguel Dora, Walter Escouto Machado, Vânia A. Andrade, Rafael Selbach Scheffel, Ana Luiza Maia

**Affiliations:** Thyroid Section, Endocrine Division, Hospital de Clínicas de Porto Alegre, Ramiro Barcelos 2350, 90035-003 Porto Alegre, RS, Brazil

## Abstract

*Objective*. It is generally accepted that higher doses of radioiodine (^131^I) improve cure rates in Graves' disease (GD). In this trial we sought to evaluate whether very high ^131^I doses increase the efficacy of treatment in severe GD. *Design*. Clinical trial with historical control. Patients with GD and a goiter ≥48 mL were eligible for the study. The patients in the contemporaneous intervention cohort were treated with 250 **μ**Ci of ^131^I/mL thyroid tissue, corrected by 24-RAIU values (Group 1; *n* = 15). A subgroup of patients with GD and a goiter ≥48 mL who were treated with 200 **μ**Ci of ^131^I/mL/24-RAIU in a previously published randomized controlled trial served as a historical control group (Group 2; *n* = 15). The primary outcome evaluated was the one-year cure rate. *Results*. There were no significant baseline differences regarding age, gender, body mass index, smoking status, pretreatment with methimazole, thyroid volume, or thyroid hormone levels of the two treatment groups. The cumulative 12-month cure rate for the patients in Group 1 was 66.6%, a figure similar to the 12-month cure rate observed in Group 2 (60.0%; *P* = 0.99). *Conclusions*. Our results suggest that increasing the ^131^I dose does not improve cure rates in severe GD. This trial is registered with ClinicalTrials.gov NCT01039818.

## 1. Introduction

Graves' disease (GD) is the most frequent cause of hyperthyroidism, primarily affecting women aged 40–60 years. Radioiodine (^131^I) was introduced as a treatment in 1941 and, because of its safety, low cost, and rapid effect, has become a cornerstone in the treatment of hyperthyroidism due to GD [1]. Despite these advantages, treatment failure occurs in approximately 15–25% of patients treated with ^131^I [2–6].

Therapeutic ^131^I regimens for GD are highly variable and include a wide range of ^131^I doses [7]. Radioactive iodine can be administered using fixed or calculated doses that are based on the size of the thyroid gland and 24-hour radioiodine uptake values [24 h-RAIU]. The current guidelines for the management of hyperthyroidism recommend a high ^131^I dose of 150 to 200 *μ*Ci of ^131^I/mL thyroid tissue corrected by a 24-RAIU value (typically 10 to 15 mCi) sufficient to cause hypothyroidism, which can be accomplished equally well with either a fixed or individualized dose regimen [7]. However, even high doses of ^131^I result in a 40% failure rate in patients who present with severe hyperthyroidism, which is characterized by a large goiter, a high 24 h-RAIU value (>70%), and very high levels of thyroid hormones (T3 levels >500 ng/mL) [4]. 

Data from retrospective observational studies have indicated that higher ^131^I doses improve cure rates. Indeed, a recent systematic review with meta-analysis found a correlation between ^131^I dose and therapeutic success in GD patients [8]. To our knowledge, there are no published studies evaluating cure rates with different ^131^I doses in patients with GD and large goiters. Therefore, we conducted a clinical trial with a historical control to evaluate whether very high doses of ^131^I would increase treatment efficacy in patients with severe GD and a large goiter. 

## 2. Materials and Methods

### 2.1. Design Rationale

The database from a previous published randomized controlled trial (RCT) that included 61 GD patients treated with 200 *μ*Ci of ^131^I/mL/24 h-RAIU was used to compare the clinical characteristics of patients with successful treatments with the clinical characteristics of patients experiencing treatment failure [4]. More than 20 clinical variables from the patients' medical history, physical examination, laboratory, and thyroid ultrasound data were analyzed. Logistic regression analysis was performed to identify the independent predictors of treatment failure after radioactive iodine therapy. Only those variables with less than 5% of the data missing and *P* values <0.10 in the univariate analysis were included in the multivariate model.

This approach identified thyroid goiter volume (*P* = 0.006) as the strongest predictor of ^131^I treatment failure. The cut-off point for this variable was determined by looking for the best discriminatory value in the individual receiver operator characteristic (ROC) curve. Thyroid volumes ≥48 mL displayed a sensitivity of 67% and a specificity of 84%; patients with a goiter volume ≥48 mL showed treatment failure rates of 40.0%, while patients with smaller goiters exhibited treatment failure rates of only 6.5% (*P* = 0.005, unpublished data). Thus, we aimed to evaluate whether increasing ^131^I doses in patients with GD and a large goiter would increase treatment efficacy.

### 2.2. Subjects

Patients with GD and a goiter ≥48 mL were eligible to enter the study. Graves' hyperthyroidism was diagnosed on the basis of suppressed TSH levels, elevated serum thyroid hormone levels, high 24 h-RAIU values, and detectable anti-TSH receptor antibody levels. The exclusion criteria included previous ^131^I treatment or thyroidectomy, signs of moderate or severe ophthalmopathy, severe heart disease, debilitating conditions, and goiters that were >150 mL or exhibited compressive symptoms. 

The contemporaneous intervention group included 15 patients who were treated with 250 *μ*Ci of ^131^I/mL thyroid tissue, corrected by the 24 h-RAIU (Group 1). A subgroup of patients with GD and goiter ≥48 mL who were treated with 200 *μ*Ci of ^131^I/mL/24 h-RAIU in a randomized controlled trial run at our institution between February 1997 and March 2000 [4] served as a historical control (Group 2, *n* = 15). 

In all patients thyroid ultrasonography (US) and 24 h-RAIU were performed at baseline by examiners blinded to medical records and biochemical data. US examination was performed with a high frequency 7.5 MHz linear array transducer (Aloka SSD-4000, Aloka Co., Ltd. Tokyo, Japan) and thyroid volume calculated applying the volumetric ellipsoid method (height × width × depth × correction factor 0.524). 24 h-RAIU was measured in a gamma counter, 24 hours after the ingestion of 5 *μ*Ci (185 kBq) of ^131^I (reference range 15–35%). 

### 2.3. Treatment Protocol and Followup

The clinical and laboratory assessments for both groups were performed on the day of ^131^I treatment; days 7, 14, and 30 after treatment; and then monthly for 1 year after treatment. Those patients on methimazole therapy received the ^131^I dosing 4 days after discontinuing the drug. The primary outcome evaluated was the one-year cure rate. Successful therapy was defined as euthyroidism or permanent hypothyroidism based on the free thyroxine (FT4) measurements obtained at each monthly visit. To avoid misclassifying each patient's thyroid status, we used 2 consecutive serum FT4 measurements in the normal or low range or, in those cases with borderline upper range values, 3 serum FT4 values. The cure date (in months) was accepted as the visit when the first serum FT4 measurement reached and persisted in the normal or low range. Therapy failure was defined as the need to repeat ^131^I treatment or the persistence of elevated thyroid hormone levels 1 year after ^131^I administration. The study protocol was approved by the ethics committee at the hospital, and all of the patients provided written informed consent. The study was registered in the ClinicalTrials.gov system under the code NCT01039818.

### 2.4. Statistical Analysis

The baseline characteristics of the two groups were compared using a *χ* ^2^ test or Fisher's exact test for qualitative variables or a *t*-test or Mann-Whitney *U* test for quantitative variables. The differences in the cumulative cure rates of the groups were also tested by Kaplan-Meier curves; comparisons between nonremission curves were performed using the Breslow test. A two-sided *P* value of less than 0.05 was considered statistically significant. The Statistical Package for the Social Sciences 17.0 (SPSS, Inc., Chicago, IL) was used for statistical analysis.

## 3. Results

The baseline characteristics of the 30 patients in the two treatment groups are shown in Table 1. Except for ^131^I dose, there were no significant differences between the two groups with respect to any of the clinical or laboratorial characteristics evaluated. 

**Figure 1:**
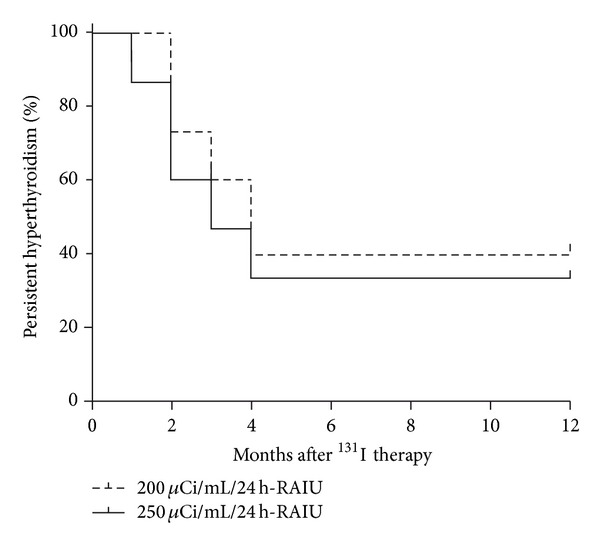
Kaplan-Meier curves indicating the responses of the two study groups to a single dose of ^131^I. The time to hyperthyroidism control was assessed by the Breslow test (*P* = 0.40).

Despite the 25% increase in calculated ^131^I dose, the cumulative 1-year cure rate for the patients in the contemporaneous intervention (Group 1) was virtually identical to the 1-year cure rate observed in the patients from the historical control group (66.6% versus 60.0%; *P* = 0.99). Moreover, the Kaplan-Meier estimates of cumulative cure rates yielded similar curves for the two treatment groups, with no differences in the time to achieve cure. Also, there were no differences in the incidence of hypothyroidism (40.0% versus 33.3%; *P* = 1.00) or euthyroidism (26.6% versus 26.6%; *P* = 1.00) at 1 year for Group 1 and Group 2, respectively. Interestingly, all of the patients were cured within four months after receiving ^131^I, indicating that this time frame is sufficient to determine if the patient responded to the first ^131^I dose (Figure 1).

Next, we compared the patients based on response to ^131^I therapy. There were no differences in the thyroid hormone levels, 24 h-RAIU rate, thyroid volume, ^131^I dose, or clinical variables of the patients. Interestingly, those patients who remained hyperthyroid were older than the patients who were cured by ^131^I therapy (39.2 ± 7.3 years versus 30.7 ± 8.2 years, resp., *P* = 0.008) (Table 2). 

## 4. Discussion

It is generally accepted that higher ^131^I doses improve cure rates in patients with GD. This study did not identify differences in the one-year cure rates of patients treated with increased doses of ^131^I (250 versus 200 *μ*Ci of ^131^I/mL thyroid tissue, corrected by 24 h-RAIU). 

Because the ^131^I-induced control of Graves' hyperthyroidism is dependent on cell damage by beta-radiation, it is biologically plausible to speculate that higher ^131^I doses would result in increased cure rates [1]. Accordingly, several retrospective and observational studies have identified correlations between higher doses and cure rates [9–12]. The effectiveness of different ^131^I doses in GD was also evaluated in two prospective clinical trials. One RCT with 59 participants compared two fixed ^131^I doses (10 versus 5 mCi) in GD, identifying increased cure rates in the group that received the higher ^131^I dose (cure rates of 88.5 versus 48.5% for 10 versus 5 mCi, resp.) [13]. However, another RCT that compared four different ^131^I regimens in 88 GD patients (low fixed [~6 mCi] versus high fixed [~9 mCi] versus low adjusted [80 *μ*Ci/g] versus high adjusted [120 *μ*Ci/g] doses) found no differences in cure rates between the groups [6]. 

Here, we used a nonparallel design to conduct a clinical trial with a historical control to evaluate whether increasing the ^131^I dose would increase cure rates in severe GD. Because the historical control group had already received a high ^131^I dose, we attempted to enhance cure rates in the contemporaneous intervention group by further increasing the ^131^I dose (very high dose). However, no differences were observed in either the time to achieve hyperthyroidism control or the cure rates of the two groups. Indeed treatment failures were observed in 11/30 (37%) of patients, and euthyroidism in 8/19 (42%) of those cured by ^131^I. The low rates of hypothyroidism (11/30, 37%) could be explained by the iodine resistance displayed by the severe Graves' disease population. It is interesting to note that, in contrast to this study, the two above-mentioned RCTs utilized treatment regimens with considerably lower ^131^I doses and were not focused on the subgroup of patients with severe GD [13]. These differences notwithstanding the results from the Leslie et al. study, are of particular interest when interpreting our data, as the GD patients included in their study also displayed large goiters (median thyroid volume of 65 mL). Thus, the results from Leslie et al. also indicate that increasing the ^131^I dose in severe GD patients does not seem to be an option for overcoming disease severity and increasing cure rates. Other strategies, such as the use of adjuvant lithium, may be considered, despite conflicting data in the literature [14, 15].

It is currently recommended that those GD patients who are not cured by an initial treatment with ^131^I should receive a second dose of ^131^I 6 months following the first dose [7]. Interestingly, we observed that all of the patients who responded to ^131^I administration were cured within four months of treatment, suggesting that the time frame for response evaluation could be shortened to 4 months in those patients that receive high ^131^I doses.

Nevertheless, our study has some limitations that should be acknowledged. Despite the fact that we used the same protocol in the two groups, the non-parallel design could lead to measurement bias. We overcame this limitation by using a well-defined outcome (cure in one year). Another possible bias could arise from the small sample size, constituted by 30 patients (23 women and 7 men). With our sample size and an *α* error of 0.05, we had a power of 82% to detect a 30% absolute difference between groups. Moreover, our results (i.e., the Kaplan Meier curve) support the argument that a larger sample size is unlikely to identify clinically significant differences in the cure rates of the two groups. 

## 5. Conclusions

In summary, the results of this clinical trial indicate that higher doses of ^131^I do not overcome disease severity and do not improve cure rates in patients with severe GD and large goiters. Future research should focus on identifying additional approaches aimed at improving the radioiodine cure rates in this subgroup of patients.

## Figures and Tables

**Table 1:** Baseline characteristics of patients with Graves' hyperthyroidism and a thyroid volume ≥48 mL, according to study group.

	Group200 *μ*Ci/mL/RAIU (*n* = 15)	Group 250 *μ*Ci/mL/RAIU (*n* = 15)	*P*
Sex (F/M)	12/3	11/4	1.000
Age (years)	34.5 ± 6.3	33.2 ± 10.9	0.697
BMI (Kg/m²)	22.9 ± 3.5	22.7 ± 3.3	0.903
Smokers [*n* (%)]	8 (53)	5 (33)	0.462
Methimazole [*n* (%)]	4 (27)	9 (60)	0.139
OC [*n* (% of females)]	6 (50)	5 (45)	1.000
Thyroid volume (mL)	59.5 ± 12.0	64.2 ± 11.7	0.281
24 h-RAIU (%)	78.9 ± 17.3	76.9 ± 11.2	0.719
Basal T4 (nmol/L)	341 ± 121	346 ± 112	0.897
Basal FT4 (pmol/L)	59 (44–89)	60 (49–95)	0.217
Basal T3 (nmol/L)	9.3 (6.0–11.5)	9.6 (5.8–12.9)	0.547
^131^I dose (mCi)	16.5 ± 4.2	20.7 ± 3.9	0.008

The values are the mean ± SD or median (25–75%). BMI: body mass index; OC: oral contraceptive; 24 h-RAIU: 24-hour radioiodine uptake.

The reference ranges for laboratory values are T4, 56.3–160.9 nmol/Liter (4.5–12.5 *μ*g/dL); free T4, 8.4–23.2 pmol/Liter (0.6–1.8 ng/dL); and T3, 1.19–2.8 nmol/Liter (78–182 ng/dL).

To convert T4 values to micrograms per dL and free T4 values to nanograms per dL, values should be divided by 12.87. To convert T3 values to nanograms per dL, values are divide by 0.01536.

**Table 2:** Baseline characteristics of patients with Graves' hyperthyroidism and a thyroid volume ≥48 mL, grouped by response to ^131^I therapy.

	Cure (*n* = 19)	Failure (*n* = 11)	*P*
Sex (F/M)	14/5	9/2	1.000
Age (years)	30.7 ± 8.2	39.2 ± 7.3	0.008
BMI (Kg/m²)	22.5 ± 3.0	23.3 ± 4.0	0.534
Smokers [*n* (%)]	9 (47)	4 (36)	0.708
Methimazole [*n* (%)]	8 (42)	5 (45)	1.000
OC [n (% of females)]	9 (64)	2 (22)	0.080
Thyroid volume (mL)	61.1 ± 12.5	63.1 ± 11.3	0.656
24 h-RAIU (%)	77.2 ± 11.2	79.1 ± 19.2	0.771
Basal T4 (nmol/L)	355 ± 127	324 ± 91	0.493
Basal T4L (pmol/L)	80 ± 31	63 ± 28	0.188
Basal T3 (nmol/L)	8.7 (5.6–10.8)	10.0 (9.1–14.0)	0.121
^131^I dose (mCi)	18.3 ± 4.2	19.0 ± 5.2	0.675

The values are the mean ± SD or median (25–75%). BMI: body mass index; OC: oral contraceptive; 24 h-RAIU: 24-hour radioiodine uptake.

The reference ranges for laboratory values are T4, 56.3–160.9 nmol/Liter (4.5–12.5 *μ*g/dL); free T4, 8.4–23.2 pmol/Liter (0.6–1.8 ng/dL); and T3, 1.19–2.8 nmol/Liter (78–182 ng/dL).

To convert T4 values to micrograms per dL and free T4 values to nanograms per dL, values should be divided by 12.87. To convert T3 values to nanograms per dL, values should be divide by 0.01536.
